# SPAG6 overexpression decreases the pro-apoptotic effect of daunorubicin in acute myeloid leukemia cells through the ROS/JNK MAPK axis in a GSTP1-dependent manner

**DOI:** 10.3389/fphar.2024.1390456

**Published:** 2024-10-23

**Authors:** Jie Luo, Li Ding, Shirui Pan, Jing Luo, Haiqiu Zhao, Jiaxiu Yin, Rong Su, Jiamin Zhang, Lin Liu

**Affiliations:** ^1^ Department of Hematology of the First Affiliated Hospital of Chongqing Medical University, Chongqing, China; ^2^ Department of Hematology, The Affiliated Hospital of Southwest Medical University, Luzhou, China

**Keywords:** AML, SPAG6, GSTP1, ROS, daunorubicin, apoptosis

## Abstract

**Introduction:**

As a malignant hematological disease, the incidence of acute myeloid leukemia (AML) has exhibited an upward trend in recent years. Nevertheless, certain limitations persist in the treatment of AML. Sperm-associated antigen 6 (SPAG6) has been implicated in the onset and progression of various human cancers, with its expression levels significantly elevated in AML. Consequently, we undertook a series of experiments to investigate the role and underlying mechanisms of SPAG6 in AML cell lines.

**Methods:**

In the *in vitro* experiments of this study, DEPs and GO and KEGG enrichment analysis subsequent to SPAG6 down-regulation were detected by TMT. CCK8 was employed to determine cell viability. The levels of apoptosis and ROS were measured by flow cytometry. In the *in vivo* experiments, a xenografted tumor model was constructed, and the expression of SPAG6 and GSTP1 in tumor tissues was detected by IHC.

**Results:**

Ultimately, our findings indicated that over-expression of SPAG6 promoted cell growth and decreased reactive oxygen species (ROS) and malondialdehyde levels. Furthermore, SPAG6 knockdown was found to diminish mitochondrial membrane potential and facilitate cell apoptosis. *In vivo*, SPAG6 could also promote tumor growth, suggesting that SPAG6 may serve as a pro-tumor factor. In addition, daunorubicin (DNR) may cause oxidative stress and initiate apoptosis, resulting in oxidative damage to AML cells. However, the overexpression of SPAG6 may attenuate the efficacy of DNR. This was due to SPAG6 promoted GSTP1 expression, thereby reducing ROS levels. Simultaneously, the elevation of GSTP1 and JNK complex may reduce the expression of p-JNK and inhibit the activation of JNK pathway, which might inhibit cell apoptosis.

**Discussion:**

In conclusion, our experiments suggested that upregulated SPAG6 might mitigate the pro-apoptotic effects of DNR through ROS/JNK MAPK axis in a GSTP1-dependent manner.

## 1 Introduction

Acute myeloid leukemia (AML) is a malignant clonal hematological disease characterized by the clonal expansion of bone marrow blast cells in peripheral blood, bone marrow, and other tissues (Shimony et al., 2023), resulting in anemia, bleeding, infection and a certain probability of extramedullary invasion (Falini and Martelli, 2023). Currently, the precise etiology of AML remains unclear, and present research indicates the potential association with genetic factors, the environment, ionizing radiation, chemical toxins, and viral infections (Yoshizato et al., 2017; Bullinger et al., 2017). Furthermore, there are indications that certain unidentified genes may contribute to the pathogenesis of patients with AML. The current therapeutic approaches are constrained and only 30% of patients receiving conventional chemotherapy achieve long-term disease-free survival, and majority of patients develop primary drug resistance or relapse (Bhansali et al., 2023; Döhner et al., 2010). Therefore, there is an urgent necessity to identify strategies aimed at enhancing the therapeutic efficacy for patients with AML. Sperm-associated antigen 6 (SPAG6), also known as Repro-SA-1, is a microtubule-associated protein (Neilson et al., 1999). It is involved in a series of biological functions, such as regulating the growth, morphology, and migration of fibroblasts. As a member of the cancer testis antigen family, SPAG6 is implicated in the initiation and progression of various human malignancies. For instance, previous studies have indicated that SPAG6 is significantly overexpressed in ovarian cancer (Coan et al., 2019), breast cancer (Siliņa et al., 2011), kidney renal papillary cell carcinoma (Li et al., 2024) and osteosarcoma. Moreover, SPAG6 may also be associated with metastasis, the Enneking stage, and the pathological grade of osteosarcoma (Bao et al., 2022). Conversely, SPAG6 expression is reduced in some tumors, such as lung cancer, owing to promoter methylation. These findings indicate that the expression of SPAG6 might be dependent on the specific tumor type.

In the context of hematological malignancies, Steinbach et al. (2006), Steinbach et al. (2015) verified that SPAG6 expression was increased in patients with AML, whereas SPAG6 expression returned to normal levels in patients with sustained complete remission. Moreover, Mu et al. (2022) reported that SPAG6 was highly expressed in AML and promoted its progression through the epidermal growth factor receptor (EGFR) family in a myosin 1D (MYO1D)-dependent manner. Besides, Ding et al. (2022) also found that SPAG6 was highly expressed in myeloproliferative neoplasms (MPN),acute lymphoblastic leukemia (ALL), myelodysplastic syndrome (MDS). Besides, SPAG6 might be related to the increase in STAT1 expression as well as the attenuation of the sensitivity to interferon-alpha (Ding et al., 2023). In our study, the upregulation of SPAG6 was found to facilitate cell proliferation and tumor growth. Furthermore, SPAG6 mitigated cellular oxidative stress and inhibited the activation of the c-Jun N-terminal kinase (JNK) pathway by promoting the expression of GSTP1. Activation of the JNK pathway promoted cell apoptosis via cytochrome C (mitochondria)/caspase3. Thus SPAG6 upregulation may attenuated daunorubicin (DNR)-induced apoptosis. Therefore, an enhanced understanding of SPAG6 may provide new insights for AML diagnosis and treatment.

## 2 Methods and materials

### 2.1 Cell culture and reagents

The human leukemia cell lines HEL, THP-1, and HL60 were obtained from the cell bank of the Type Culture Preservation Committee of the Chinese Academy of Sciences, and MV4-11 cells were sourced from Zhong Qiao Xin Zhou Biotechnology. All cell lines underwent authenticated through short-tandem repeat profiling. HEL, THP-1 and MV4-11 cells were cultured in RPMI-1640 medium (Procell) containing 10% fetal bovine serum (PAN), and HL60 cells were cultured in IMDM (Procell) containing 20% fetal bovine serum. All cells were incubated at 37°C with 5% CO_2_. DNR, SP600125 and N-acetylcysteine (NAC) were acquired from MedChem Express. The lentiviral vectors and siRNA were purchased from Gene Pharma. Transfection was performed according to the manufacturer’s instructions.

### 2.2 Cell counting kit 8 (CCK-8) assay

Cells from each group (5 × 10^3^) were seeded in 96-well plates containing 100 µL medium and cultured in incubators. At time points of 0, 24, 48, and 72 h, respectively, 10 µL of CCK-8 reagent was added; the absorbance at 450 nm was determined after incubation at 37°C for 2 h. Then, 1×10^4^ cells from each group were seeded in 96-well plates containing 100 µL medium. The cells were treated with DNR at different concentrations for 48 h. Subsequently, 10 µL of CCK-8 reagent was added and incubated at 37 °C for 2 h to measure the OD value. And cell viability was calculated according to the OD value.

### 2.3 Western blot analysis

Cells in each group were collected, RIPA and PMSF were utilized to extract the protein, BCA was used to detect the protein concentration, and 35 µg protein was calculated according to the concentration. SDS-PAGE was performed and the proteins were transferred to a polyvinylidene fluoride (PVDF) membrane. The membranes were blocked with 5% skim milk for 2.5 h at room temperature. After that the primary antibodies were added and incubated overnight at 4°C. The primary antibodies used were against SPAG6 (Cat No. 12462-1-AP; Proteintech, China), GSTP1 (Cat No. 15902-1-AP; Proteintech, China), Caspase3 (Cat No. AF300113; Aifang Biological, China), Cleaved caspase3 (Cat No. AF00006; Aifang Biological, China), JNK (Cat No. 66210-1-Ig, Proteintech, China), p-JNK (Cat No. 80024-1-RR; Proteintech, China), and β-actin (Cat No. bs-10966R; Bioss, China) at a dilution of 1:1,000. HRP-conjugated Affinipure Goat Anti-Mouse IgG (Cat No. bs-0296G, Bioss, China) or Goat Anti-Rabbit IgG (Cat No. bs-80295G-HRP; Bioss, China) were employed as secondary antibodies at a dilution ratio of 1:500. All protein bands were detected using an ECL kit (Advansta, United States). All tests were repeated thrice.

### 2.4 Cell apoptosis and ROS detection

Cells in each group were collected by centrifugation, re-suspended in PBS, and centrifuged at 1,000 rpm for 5 min twice. Finally, the cells were re-suspended in 300 µL PBS. An Annexin V-fluorescein isothiocyanate/propidium iodide (PI) kit and DCFH-DA were respectively utilized to detect apoptosis and ROS. Flow cytometry was used to analyze the stained cells immediately.

### 2.5 Mitochondrial membrane potential detection

1 × 10^6 cells were collected and re-suspended in 1 mL of RPIM-1640, at the same time 1 µL of 1,000 × TMRE solution was added. Subsequently, incubated at 37°C for 25 min and washed twice with PBS. Then 1 mL RPIM-1640 containing 10 µL of Hoechst33342 dye was added for staining for 10 min. Finally, cells were seeded in 6 well, and observed the fluorescence of TMRE and Hoechst under a microscope.

### 2.6 Malondialdehyde (MDA) analysis

Cells from each group were collected, and RIPA buffer was added to lyse the cells for 30 min, followed by centrifugation for 15 min at 12,000 rpm/min. The supernatant was then collected, and the protein concentration was detected using a BCA assay. According to the protocol of the Lipid Peroxidation Malondialdehyde Assay Kit, 200 µL MDA working solution was added into the supernatant. The reaction was incubated at 100°C for 10 min, and centrifuged at 1,000 *g* for 15 min. After that, 200 µL supernatant was collected and the OD value at 532 nm was determined. Finally, the MDA levels were calculated based on the protein concentration and absorbance values.

### 2.7 Tandem mass tags (TMT) analysis for SPAG6-interacting proteins

To identify SPAG6-related proteins and their biological functions, TMT analysis was performed on SPAG6 knockdown and control groups of THP-1 cells. The experiments were conducted by Novogene Technology (Beijing, China). The samples underwent protein extraction, quantification, detection, enzyme digestion, salt removal (Wu et al., 2010), labeling (for iTRAQ and TMT), enrichment of modified peptides (for modified proteome), fraction separation, and mass spectrometry detection. Subsequently, systematic bioinformatic analysis (protein function annotation) was then carried out for all identified proteins. Functional enrichment and protein interaction network analyses were performed for all differentially expressed proteins.

### 2.8 Xenograft tumor model

All animal experiments were approved by the Animal Use and Care Committee of Chongqing Medical University. Four-week-old male BALB/ca-nu mice were procured from Beijing Vital River Laboratory Animal Co., Ltd. and reared under specific pathogen-free conditions. The mice were randomly divided into 4 groups (n = 5), namely, SPAG6 overexpression and SPAG6 control of HL60, as well as SPAG6 knockdown and control of THP-1. Approximately 5 × 10^6^ cells were injected subcutaneously into mice, their weight was monitored twice a week, and the tumors were removed after 21 days.

### 2.9 Immunohistochemistry (IHC) and histological examination (HE) staining

Firstly, the tissues were embedded in wax and sectioned. Subsequently, the paraffin-embedded sections were then dewaxed, stained with HE, dehydrated, sealed, and observed under a microscope. IHC staining, additional procedures were requisite, including paraffin section dewaxing, antigen repair, blocking of endogenous peroxidase, serum blocking, primary antibody incubation, secondary antibody incubation, diaminobenzidine color development, nuclear staining, dehydration, and sealing. The primary antibodies used for IHC staining were SPAG6 (Cat No. 12462-1-AP; Proteintech, China), GSTP1 (Cat No. 15902-1-AP; Proteintech).

### 2.10 Statistical analysis

All data were analyzed using GraphPad Prism (version 8.0.1) software, and the average ±standard error in the measurement were presented. The t-test was used for statistical comparison. Statistical significance was designated at *p* < 0.05 labeled as *, and at *p* < 0.01 labeled as**. All tests were repeated at least three times.

## 3 Results

### 3.1 SPAG6 promotes proliferation and reduces oxidative stress in AML cells

**FIGURE 1:**
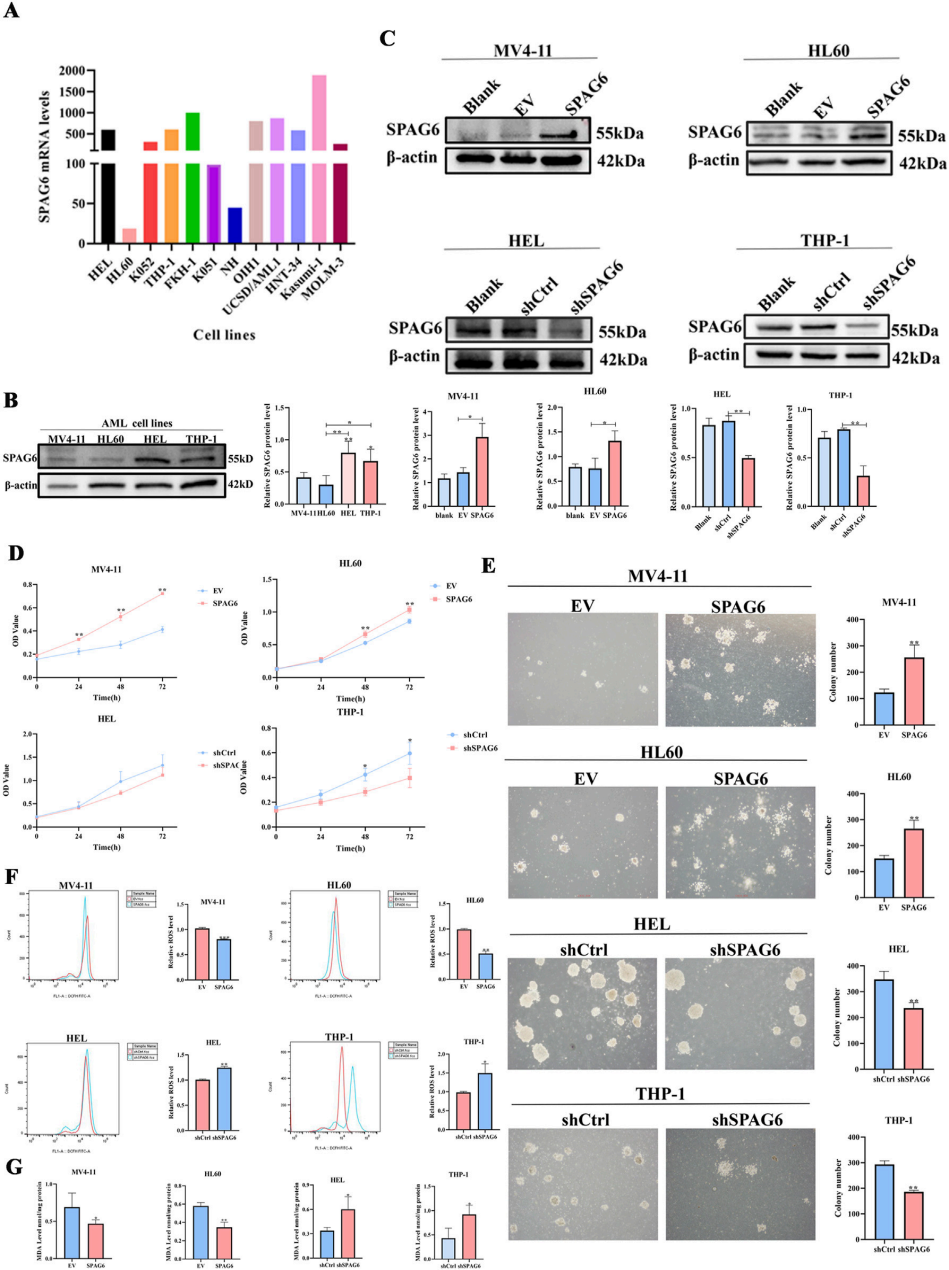
SPAG6 promotes proliferation and reduce oxidative stress in AML cells **(A)** The mRNA levels of SPAG6 in different AML cell lines were retrieved from GEO database (https://www.ncbi.nlm.nih.gov/geo/query/acc.cgi?acc=GSE35159); **(B)** Western blot analysis was conducted to determine the protein levels of SPAG6 in MV4-11,HL60,HEL and THP-1; **(C)** Lentiviral transfection was employed to establish SPAG6-overexpression (labeled SPAG6) and control (labeled EV) groups in MV4-11 and HL60 cell lines; meanwhile, HEL and THP-1 cells were transfected with SPAG6-knockdown and control lentivirals. Western blot verified the expression of SPAG6 in SPAG6 knockdown or overexpression AML cells; and western blot analysis was repeated three times. **(D)** CCK8 was used to detect the cell viability of four cell lines at 24 h, 48 h and 72 h after seeding; **(E)** Colony formation assays were conducted to detect the numbers of clones formed in AML cell lines after 14 days of seeding; **(F)** ROS levels in different groups were measured by flow cytometry following 15 min of DCFH-DA staining; **(G)** MDA Assay kit was utilized to detect the MDA levels of AML cell lines. All data were shown as the mean ± SD from three repeated experiments.*p < 0.05, **p < 0.01.

**FIGURE 2:**
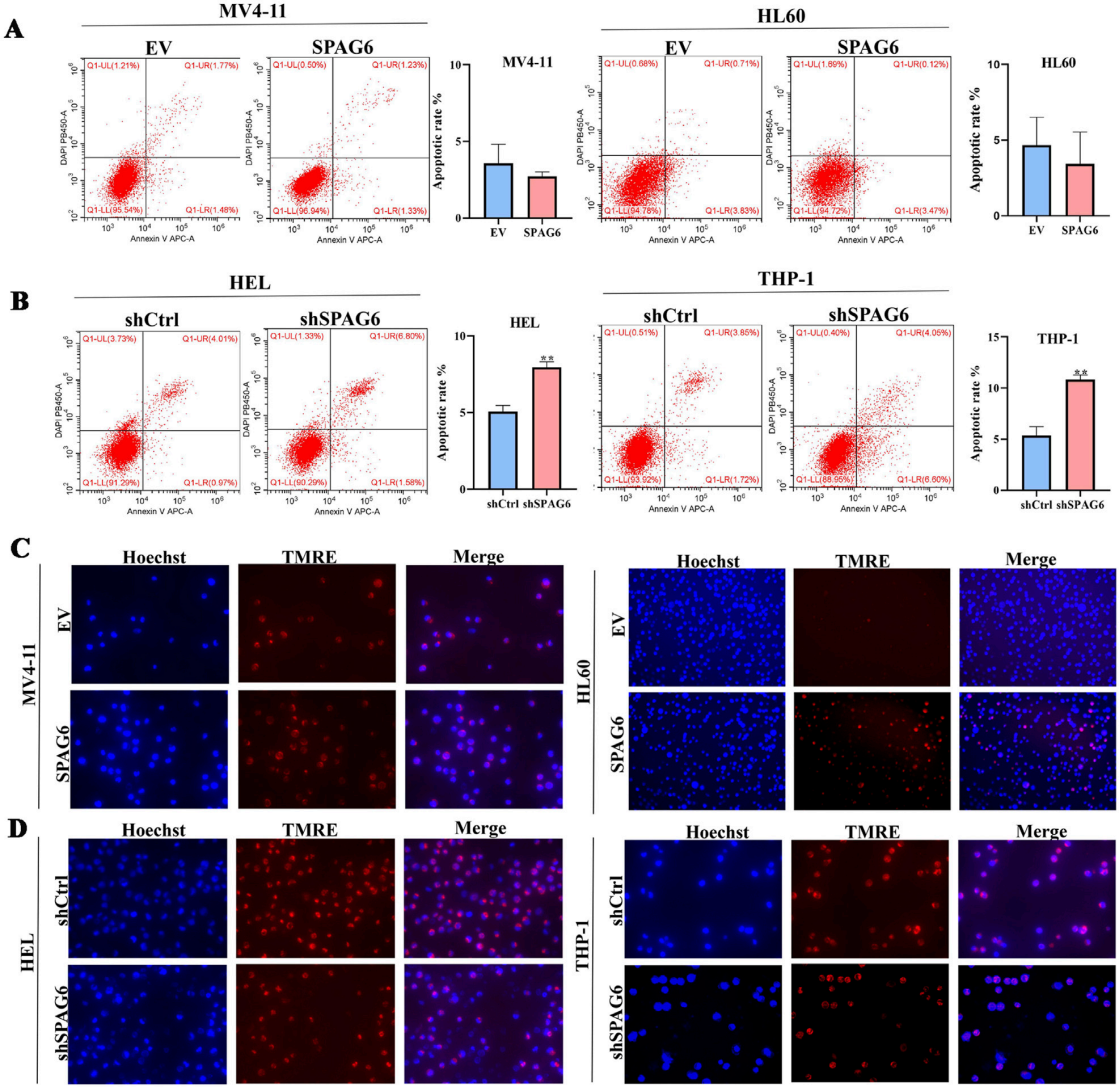
SPAG6 knockdown decreased the mitochondrial membrane potential and promoted apoptosis in AML cells. **(A, B)** cell apoptosis in different groups was analyzed by flow cytometry after Annexin V/PI staning. **(C, D)** The alterations of mitochondrial membrane potential in each group were varified by TMRE staning for 25 min and observed under a fluorescence microscope, with the scale bar being 50 μm. All data were shown as the mean ± SD from three repeated experiments. *p < 0.05, **p < 0.01.

In our study, the expression of SPAG6 in the MV4-11 and HL60 cell lines was lower than that in the HEL and THP-1 cell lines (Figures 1A, B). Consequently, we established SPAG6 overexpressing cell lines in MV4-11 and HL60, and SPAG6 knockdown cell lines in HEL and THP-1 cells (Figure 1C); EV represents an empty vector, while shCtrl serves as the negative control for short hairpin RNA. We performed a series of functional cellular experiments. Cell proliferation was measured using the CCK-8 and colony formation assays, which indicated that the cell viability and clone-forming ability of the SPAG6 knockdown group were decreased, whereas the opposite results were observed in the SPAG6 overexpression group (Figures 1D, E). In the detection of apoptosis and mitochondrial membrane potential, we discovered that the apoptosis rate of SPAG6 overexpression group was slightly decreased without statistical significance (Figure 2A), while in the SPAG6 knockdown group, the apoptosis rate was increased (Figure 2B). And the TMRE fluorescence was not significantly altered in MV4-11 cells while TMRE was enhanced in the overexperssion group compared with the control in HL60 cells (Figure 2C). However, the TMRE fluorescence intensity was attenuated, indicating that the mitochondrial membrane potential was decreased in HEL and THP-1 cells (Figure 2D). Therefore, we concluded that SPAG6 promotes AML cell growth *in vitro*. In our previous transcriptome sequencing analysis, we found that SPAG6 might be related to biological processes, such as oxidation-reduction enzyme activity and oxidation-reduction reactions; therefore, we examined the ROS and MDA in each group. As shown in Figures 1F, G, the levels of ROS and MDA were elevated in the SPAG6 knockdown group, but decreased in the SPAG6 overexpression group.

### 3.2 Effect of SPAG6 knockdown on the protein expression profile of AML cells

**FIGURE 3:**
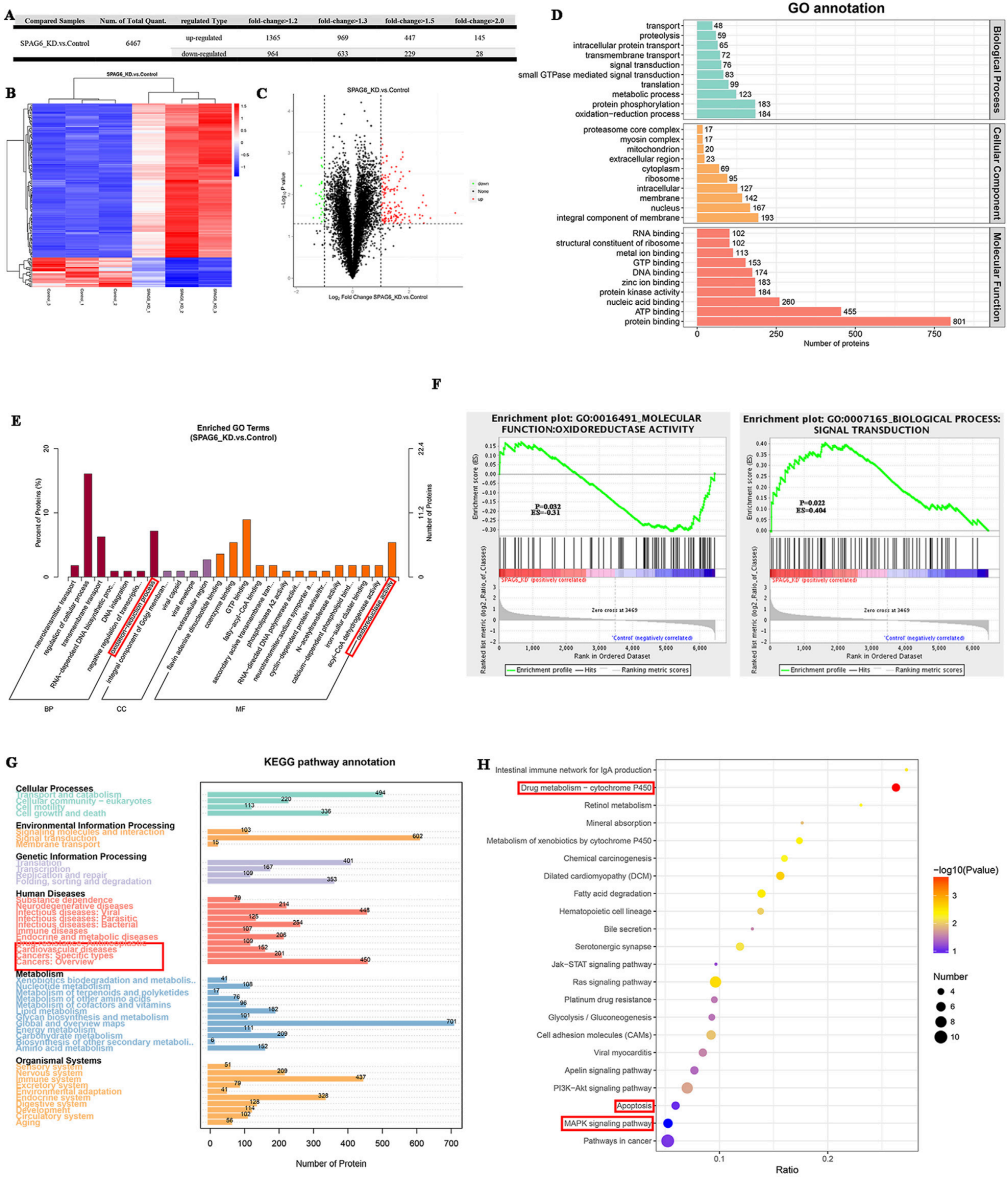
The effect of SPAG6 downregulation on the protein expression profile of AML cells. **(A)** TMT analysis was performed in both the SPAG6 knockdown and control groups of THP-1 cells (n = 3 for each, respectively). The quantity of diferentially expressed proteins (DEPs) varies according to the criterion of fold change; **(B)** Clustering heat maps of 145 upregulated proteins and 28 downregulated proteins. The vertical represents the sample cluster, the horizontal represents the protein cluster, and the shorter the cluster branch, the higher the similarity. Blue indicates downregulation and red indicates upregulation.; **(C)** Volcano maps of DEPs, where the horizontal axis represents the fold change of the DEPs (log2 value), and the vertical axis represents the *p*-value (-log10 value). Black represents non-significant proteins, red represents upregulated proteins, and green represents downregulated proteins. **(D, E)** GO annotation and GO enrichment analysis of DEPs.**(F)** GSEA analysis was performed on GO items based on the alteration of protein expression. The greater the absolute value of Erichment Score (ES), the higher the degree of enrichment. Positive ES indicates that the functional set protein is enriched in the upregulated protein, and negative ES indicates that the functional set protein is enriched in the downregulated protein; **(G, H)** KEGG pathway annotation and KEGG enrichment analysis of DEPs.

To clarify the effects of SPAG6 on biological functions, we collected shSPAG6 (n = 3) and shCtrl (n = 3) THP-1 cells for TMT analysis. In total, 6,519 proteins were identified in the six samples, among which 6,467 were quantitative proteins. According to the criterion of > 2-fold changes (upregulated more than 2.0 times or downregulated less than 0.5 times) and *p* < 0.05, we screened for significant differentially expressed proteins (DEPs) (Figure 3A). Based on this, 145 upregulated and 28 downregulated proteins were detected. The relevant DEPs clustering heat maps and volcano maps are shown in Figures 3B, C. In the GO functional annotation, we identified that proteins were associated with the oxidation-reduction process (Figure 3D), and that DEPs were enriched in oxidoreductase activity and the oxidation-reduction process (Figure 3E). Further, based on gene-set enrichment analysis (GSEA), the DEPs were enriched in oxidoreductase activity (Figure 3F). Kyoto Encyclopedia of Genes and Genomes (KEGG) analysis revealed that the detected proteins were associated with cancer and drug resistance (Figure 3G). In addition, DEPs were enriched in drug metabolism, the MAPK pathway, and cell apoptosis (Figure 3H).

### 3.3 Upregulation of SPAG6 decreases the pro-apoptotic effect of DNR by reducing oxidative stress

**FIGURE 4:**
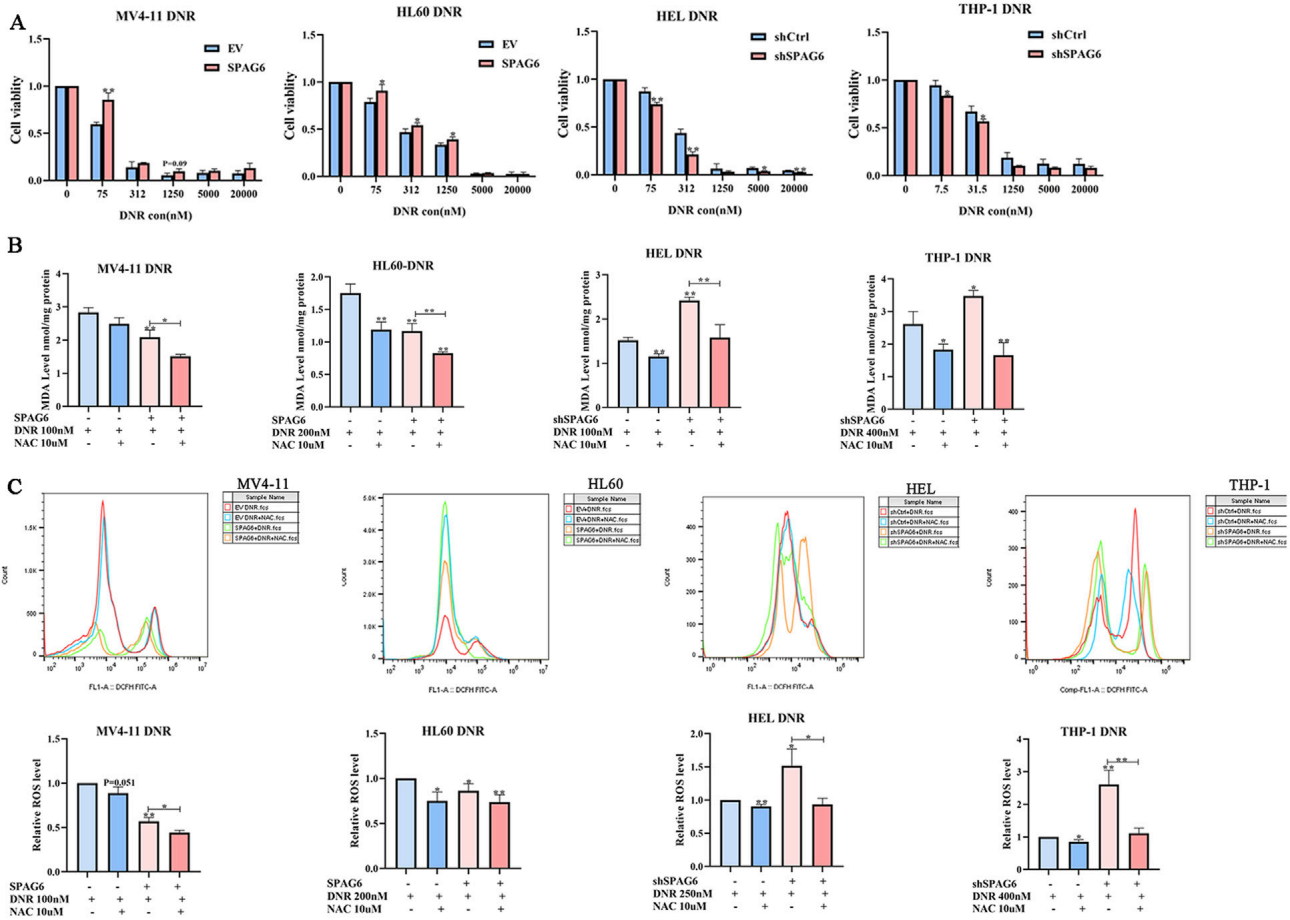
Effect of SPAG6 on cell viability after drug treatment. **(A)** CCK8 analysis was conducted to evaluate the cell viability of MV4-11,HL60,HEL and THP-1 cells treated with DNR at different concentrations (0, 75, 312, 1,250, 5,000, 20000 nM) for 48 h; MV4-11,HL60,HEL and THP-1 cells were treated with DNR at 100 nM, 200 nM, 200 nM and 400 nM respectively, with or without 10 μM NAC for 48 h. And then **(B)** MDA analysis of AML cell lines was performed. **(C)** Flow cytometry was applied to detect the ROS levels in AML cell lines, and the relative ROS levels were calculated by mean FITC-A. All data were shown as the mean ± SD from three repeated experiments.*p < 0.05, **p < 0.01.

The CCK-8 assay was employed to determine cell viability after 48 h of treatment with different concentrations of DNR. The results indicated that as the concentration of DNR increased, the cell viability of AML cells diminished. Moreover, cell viability of the SPAG6 overexpression group was higher than that of the control group in MV4-11 and HL60 cells. In contrast, cell viability of the SPAG6 knockdown group was lower than that of the control group in the THP-1 and HEL cell lines (Figure 4A). The IC50 values of DNR were presented in Table 1. These results suggested that SPAG6 may affect the cytotoxic effect on DNR in AML cell lines.

**TABLE 1:** IC50 of DNR.

Drugs		DNR (nM)
Groups		
MV4-11	EV	88.68
	SPAG6	166.6
HL60	EV	216.8
	SPAG6	1,284
HEL	shCtrl	102.1
	shSPAG6	65.48
THP-1	shCtrl	394
	shSPAG6	308.8

Subsequently, DNR at the IC50 concentration was used to treat the cell lines for 48 h. 100 nM for MV4-11 and HEL cells, 200 nM for HL60 cells and 400 nM for THP-1 cells, respectivly. And the levels of ROS and MDA were detected. We found that the levels of ROS and MDA in the SPAG6 overexpression combined with DNR treatment group were lower than those in the DNR alone group. Moreover, the levels of ROS and MDA in the SPAG6 knockdown combined with DNR treatment group were higher than those in the DNR alone group, and these effects were reduced using the antioxidant NAC (Figures 4B, C). This suggests that SPAG6 upregulation reduces DNR-induced oxidative stress.

**FIGURE 5:**
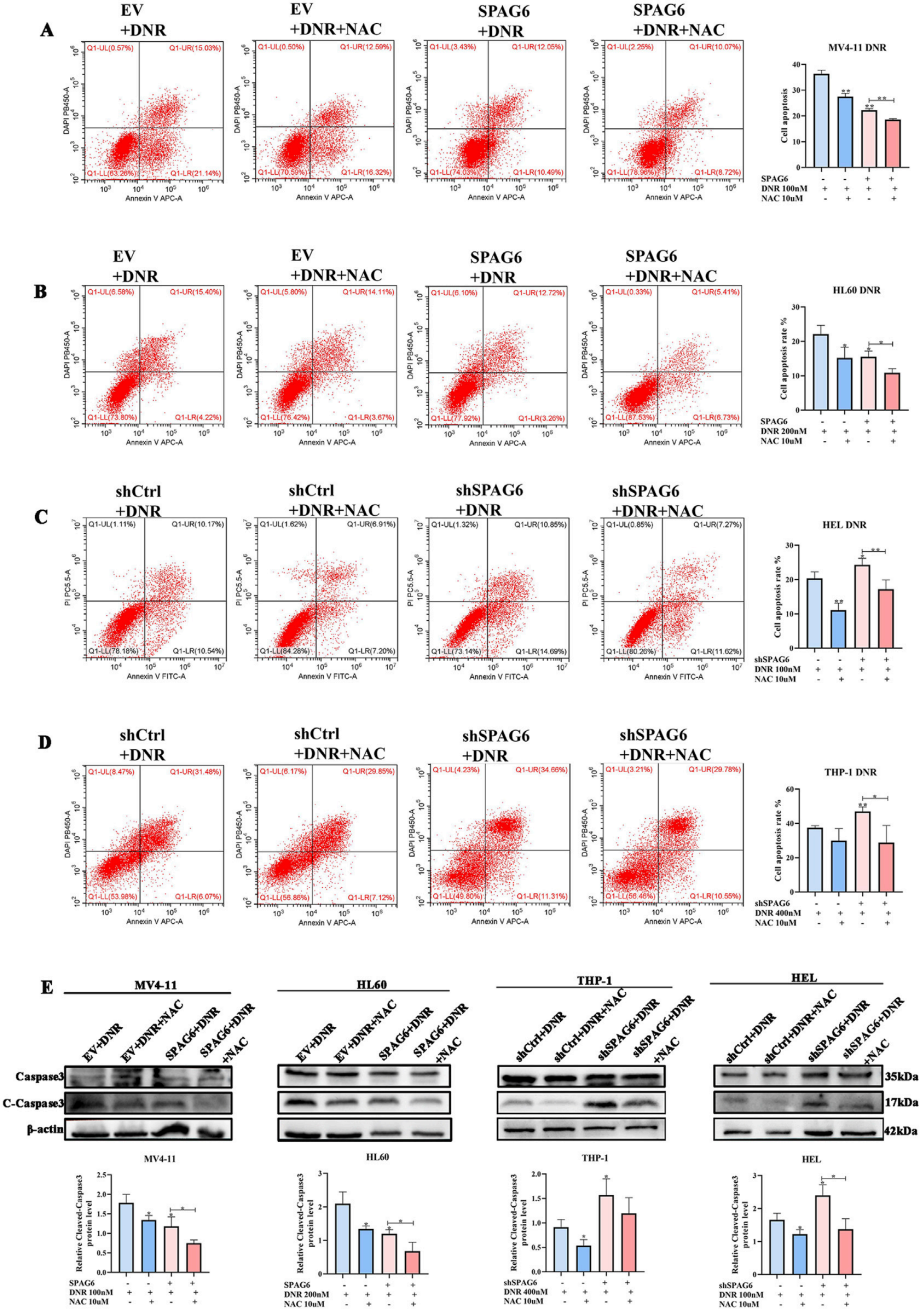
Influence of SPAG6 on the pro-apoptotic effect of DNR. **(A, B)** Apoptosis of MV4-11 and HL60 cells treated with 48-hour DNR at concentrations of 100 nM and 200 nM, respectively, with or without 10 μM NAC treatment; **(C, D)** Apoptosis of HEL and THP-1 cells treated with DNR at concentrations of 100 nM and 400 nM for 48 h, respectively, with or without 10 μM NAC; And cell apoptosis analysis was detected by flow cytometry. **(E)** The expression of Caspase 3 and Cleaved-caspase 3 and corresponding statistical analysis in each groups. Western blot analysis was repeated three times. All data were shown as the mean ± SD from three repeated experiments.*p < 0.05, **p < 0.01.

Next, we explored the apoptotic changes in DNR-treated cells; the results indicated that DNR promoted cell apoptosis. Additionally, the proapoptotic effect of SPAG6 knockdown combined with DNR was stronger than that of DNR alone (Figures 5C, D), and SPAG6 overexpression combined with DNR reduced the rate of apoptosis induced by DNR (Figures 5A, B). Notably, NAC inhibited DNR-induced apoptosis. These results were consistent with the protein changes in cleaved-caspase3, a proapoptotic indicator (Figure 5E). Therefore, we concluded that DNR may promote apoptosis by inducing oxidative stress, whereas SPAG6 upregulation may inhibit this process.

### 3.4 SPAG6 regulates DNR-induced apoptosis through the GSTP1/JNK axis

**FIGURE 6:**
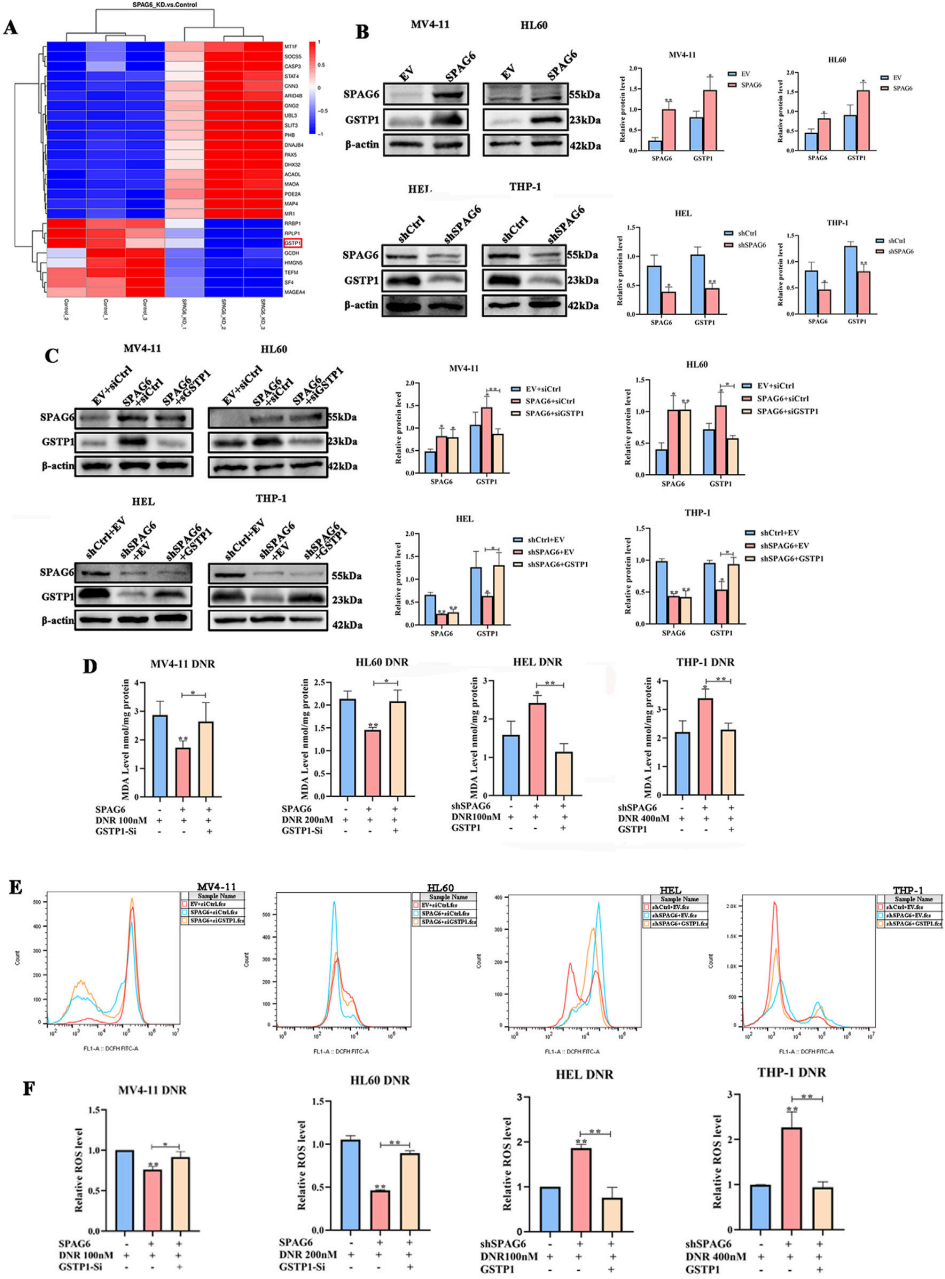
The relationship of GSTP1 and SPAG6. **(A)** Details of 20 DEPs were presented in the form of a cluster heat map; **(B)** The expression of GSTP1 and SPAG6 in AML cell lines were verified by wstern blot and repeated three times; **(C)** Small interfering RNA and lentivirus were employed to reverse the expression of GSTP1, and western blot was used to analyze the protein levels and repeated three times; MV4-11,HL60,HEL and THP-1 cells were treated with 48 h DNR treatment (100 nM, 200 nM, 200 nM and 400 nM respectively), and then **(D)** MDA assay kit was applied to measure the MDA level of each groups; **(E, F)** The relative ROS level of each groups.*p < 0.05, **p < 0.01.

**FIGURE 7:**
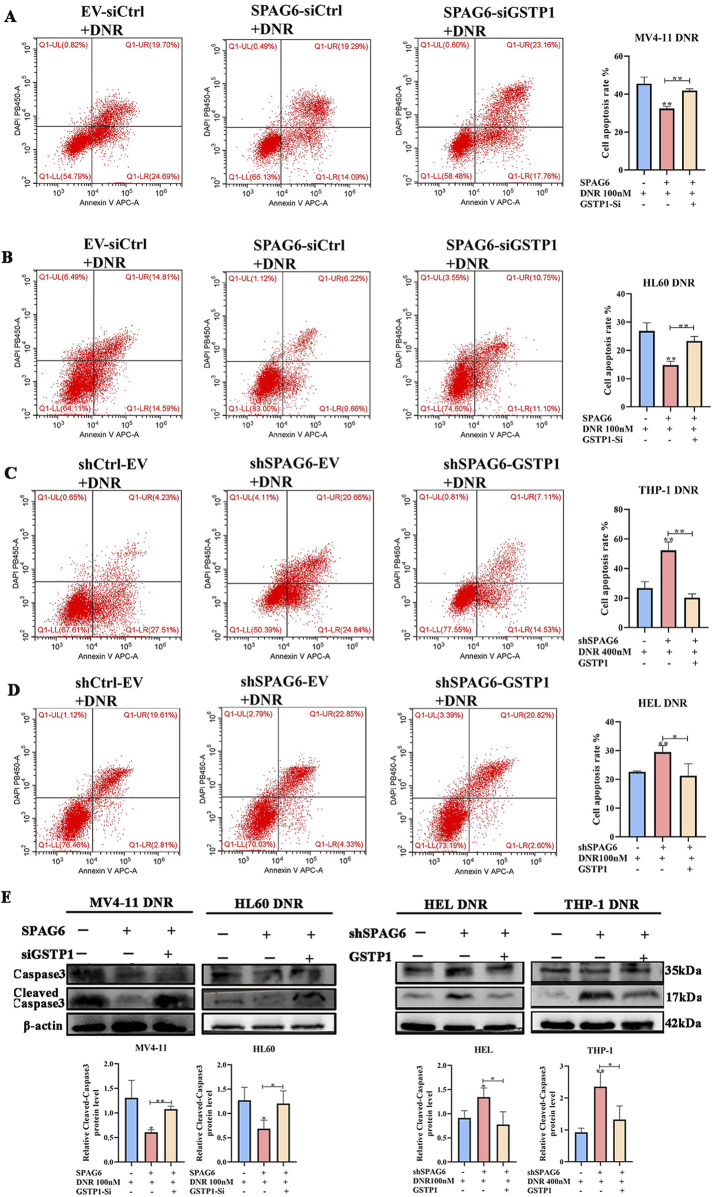
SPAG6 regulates DNR induced apoptosis in GSTP1 manner. **(A−D)** Flow cytometry was employed to determine the cell apoptosis rate. **(E)** The expression levels of Caspase 3 and Cleaved-caspase 3 in each groups were detected by western blot; All data were shown as the mean ± SD from three repeated experiments.*p < 0.05, **p < 0.01.

As mentioned above, there were 28 downregulated proteins in the DEPs, among which the expression of GSTP1 in the SPAG6 knockdown group was lower than that in the control group (Figure 6A). As GSTP1 is an antioxidant enzyme believed to be involved in drug metabolism, we probed whether SPAG6 influences DNR-induced apoptosis through GSTP1. Firstly, we analyzed the expression of GSTP1 in AML cells and found that GSTP1 expression was diminished in the SPAG6 knockdown group, whereas it increased in the SPAG6 overexpression group (Figure 6B). Consequently, small interfering RNA and lentiviruses were used to reverse GSTP1 expression (Figure 6C). After DNR treatment, we measured the levels of MDA and ROS in each group. The knockdown of GSTP1 mitigated the elevation of ROS and MDA levels induced by SPAG6 overexpression. Meanwhile, the overexpression of GSTP1 could also counteract the increase in ROS and MDA levels resulting from SPAG6 knockdown (Figures 6D–F). Therefore, SPAG6 might regulate the DNR-induced oxidative stress in a GSTP1-dependent manner. Subsequently, we determined the rate of apoptosis in each group. We found that the SPAG6+siCtrl group exhibited the lowest apoptosis in MV4-11 and HL60 cells. Furthermore, following the knockdown of GSTP1, an increase in apoptosis was noted (Figures 7A, B). On the other hand, the shSPAG6+EV group showed the highest apoptosis rate in HEL and THP cells, whereas overexpression of GSTP1 resulted in the reduction of cell apopotosis. (Figures 7C, D). In addtion, the expression levels of cleaved caspase3 were also consistent with alterations in the apoptosis rate (Figure 7E). Therefore, we believe that SPAG6 may regulate cellular oxidative stress in a GSTP1-dependent manner, thereby influencing the pro-apoptotic effect of DNR.

**FIGURE 8:**
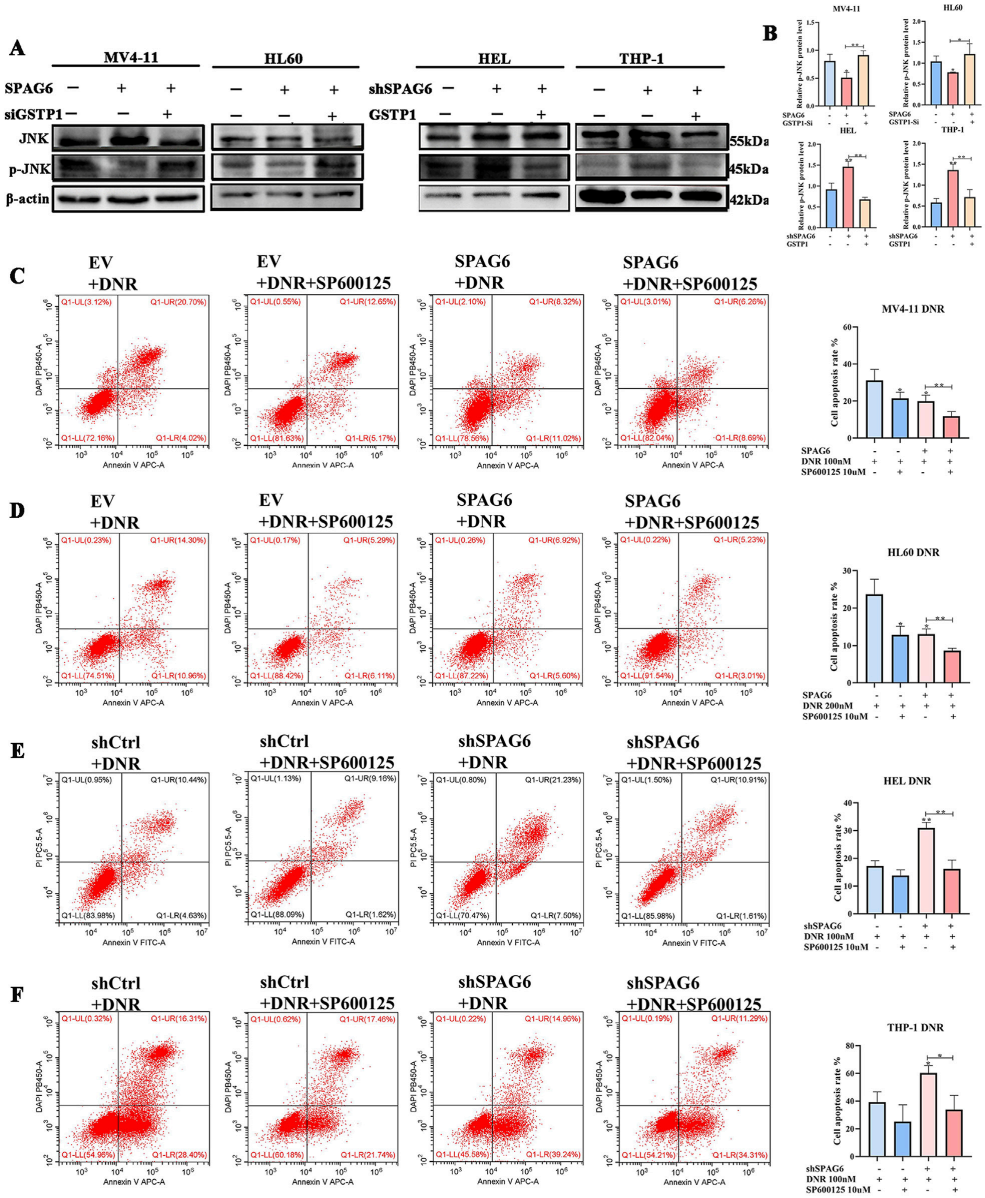
SPAG6 regulates DNR induced apoptosis in GSTP1/JNK axis. **(A, B)** Western blot and the corresponding statistical analysis were utilized to verify the expression of JNK and p-JNK in AML cell lines. Western blot analysis was repeated three times. **(C, D)** Apoptosis of MV4-11 and HL60 cells treated with DNR at 100 nM and 200 nM for 48 h, respectively, with or without 10 μM SP600125 treatment; **(E, F)** Apoptosis of HEL and THP-1 cells treated with DNR at 100 nM and 400 nM for 48 h, respectively, with or without 10 μM SP600125 treatment; And cell apoptosis analysis was detected by flow cytometry. All data were shown as the mean ± SD from three repeated experiments.*p < 0.05, **p < 0.01.

GSTP1 forms a dimer with JNK under physiological circumstances, and thus deactivates the JNK pathway. Therefore, GSTP1 is considered as a natural JNK inhibitor. In our study, p-JNK expression was elevated in the shSPAG6+EV group but decreased in the SPAG6+siCtrl group. Meanwhile, p-JNK expression was decreased in the shSPAG6+GSTP1 group and increased in the SPAG6+siGSTP1 group (Figures 8A, B). After that, we conducted flow cytometry to figure out the effect of JNK inhibitor on cell apoptosis. The results suggested that the apoptosis rate of SPAG6 overexpression combined with DNR was lower than that of EV + DNR group in MV4-11 and HL60 cells (Figures 8C, D). Besides, the shSPAG6+DNR group showed a higher rate of cell apoptosis compared to shCtrl + DNR group in HEL and THP-1 cells (Figures 8E, F). Interestingly, when we treated AML cells with JNK inhibitor SP600125 (10uM) at the same time as the DNR treatment, it was found that SP600125 was able to reduce the cell apoptosis rate in each group. These findings indicate that the JNK pathway is related to cell apoptosis, and studies have reported that the activation of JNK pathway might promote apoptosis, which is consistent with our results.

### 3.5 Upregulated SPAG6 promotes tumor growth *in vivo*

**FIGURE 9:**
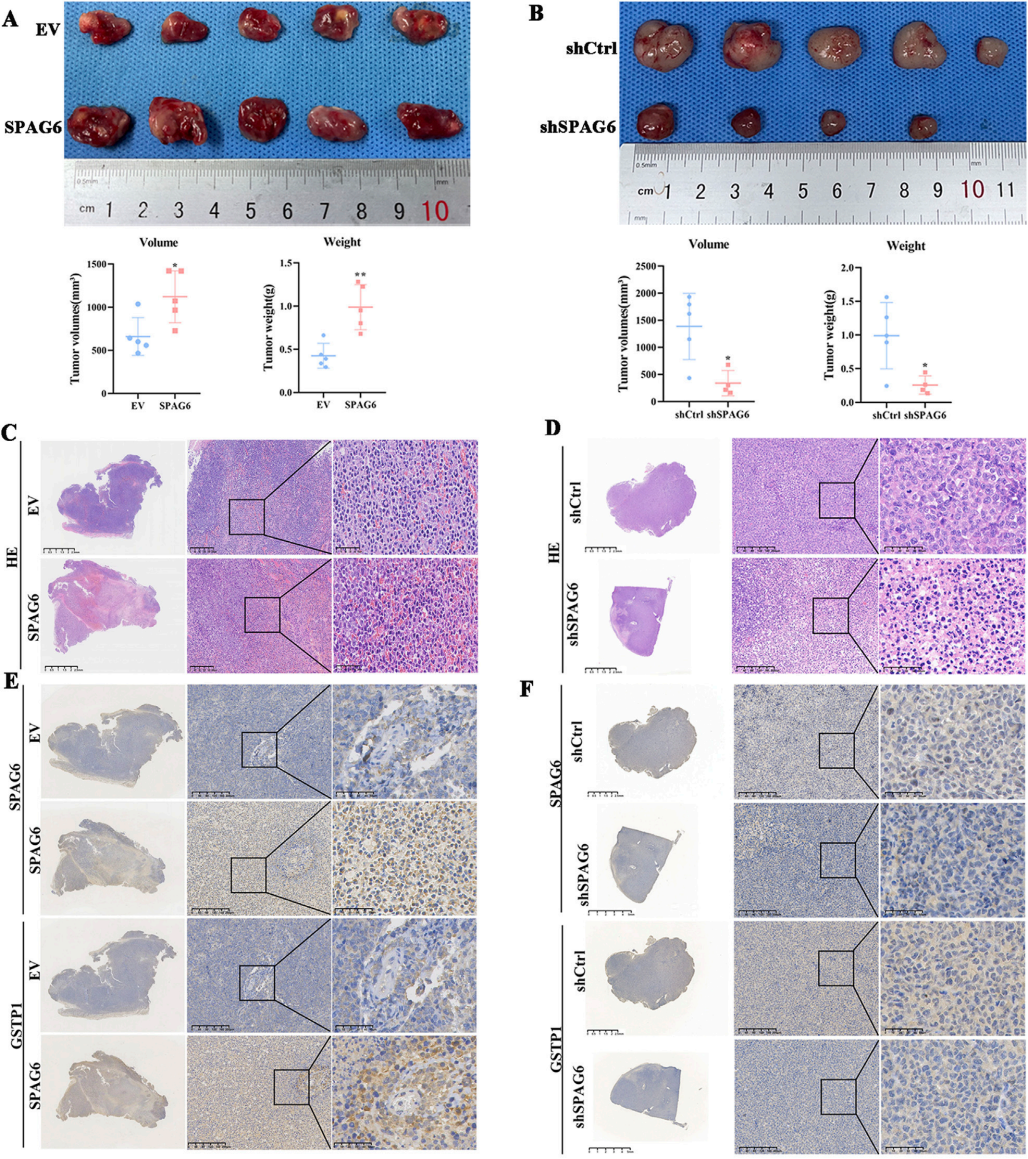
Upregulated SPAG6 promotes the tumor growth. **(A, B)** The tumor volume and weight of SPAG6 overexpression or downregulation were compared with controls respectively. The formula for volume calculation is Volume = (width^2 * length)/2; **(C, D)** Representative images of HE staining in each groups are presented. And the scale bars are 2.5 mm, 200 μm or 50 μm, respectively; **(E, F)** Representative images of IHC staining of SPAG6 and GSTP1 in each groups are shown. And the scale bars are 2.5 mm, 200 μm or 50 μm, respectively. All data were shown as the mean ± SD from three repeated experiments.*p < 0.05, **p < 0.01.

To verify the effect of SPAG6 on tumor growth *in vivo*, we selected HL60 and THP-1 cells for subcutaneous tumor formation experiments. In comparison with the control group, the tumor volume and weight increased in the SPAG6 overexpression group (Figure 9A) but decreased in the SPAG6 knockdown group, indicating that SPAG6 facilitated tumor growth *in vivo* (Figure 9B). HE staining showed more blood sinuses and higher cell density in SPAG6-overexpressing cell-derived tumors (Figure 9C), whereas some necrotic areas were observed in the SPAG6 knockdown group (Figure 9D). IHC was used to determine the expression of GSTP1 and SPAG6 in tumor tissues. Further, SPAG6 and GSTP1 were upregulated in the SPAG6 overexpression group (Figure 9E) and downregulated in the shSPAG6 group (Figure 9F).

## 4 Discussion

Currently, SPPAG6 is known to be highly expressed in diverse human malignant tumors and is involved in various biological processes (Zheng et al., 2019). Thus, SPAG6 may play a significant role in tumorigenesis and progression. For instance, the expression of SPAG6 was increased in breast cancer, osteosarcoma, and ovarian cancer, which could be associated with the pathological grade of the disease (Bao et al., 2022). In our previous study, SPAG6 was found to be highly expressed in AML, which might be related to risk stratification, prognosis, and other disease characteristics. Moreover, SPAG6 was showen to promote proliferation of AML cells both *in vivo* and *in vitro*. These findings suggested that SPAG6 plays a carcinogenic role in AML. In contrast, SPAG6 demonstrated decreased expression in lung cancer (Altenberger et al., 2017; Wu et al., 2022) and bladder cancer (Kitchen et al., 2015) owing to high methylation of its promoter (Altenberger et al., 2017), and was considered a tumor suppressor gene in these cancers.

In our previous studies, SPAG6 was found to be highly expressed in patients with AML (Mu et al., 2022) and MPN (Ding et al., 2022), and was associated with disease prognosis. Besides, SPAG6 expression was significantly elevated in patients with MDS when compared with the normal controls (Jiang et al., 2019). Additionally, SPAG6 might serve as a prognostic indicator of overall survival in patients with AML. Mechanistically, SPAG6 promoted the translocation of MYO1D from the cytoplasm to the cell membrane, thereby activating the PI3K/AKT and ERK signaling pathways, which ultimately accelerating AML cell proliferation and tumor progression. In BCR-ABL-negative MPN cases, the downregulation of SPAG6 resulted in reduced cell proliferation and increased apoptosis rate (Mu et al., 2022; Ding et al., 2022). Moreover, SPAG6 has been shown to induce apoptosis in SKM-1 cells (Yin et al., 2018; Yang et al., 2015). Zhang confirmed that SPAG6 inhibited apoptosis and promoted cell proliferation through the PTEN/PI3K/AKT pathway in Burkitt’s lymphoma (Zhang et al., 2020). In this study, SPAG6 knockdown in HEL and THP-1 cells led to an increase in apoptosis rates. Additionally, SPAG6 also affected the proliferation of MV4-11, HL60, HEL, and THP-1 cells. This is in line with the results of our previous study. Notably, the expression of SPAG6 in AML cells was correlated with alterations in ROS, MDA, and mitochondrial membrane potential. TMT analysis illustrated that differentially expressed proteins are involved in oxidoreductase activity, oxidation-reduction processes, and mitochondria. Therefore, we contend that SPAG6 may play a critical role in maintaining redox homeostasis within these cells.

The glutathione S-transferase (GST) protein family encompasses a series of detoxification isoenzymes (Aitken et al., 2006; Hayes et al., 2005),featuring significant structural similarities and a certain degree of overlapping functions (Singh and Reindl, 2021). Generally, GSTs conjugate glutathione (GSH) to exogenous compounds and inhibit oxidative stress (Lei et al., 2021; Dong et al., 2018; Mazari et al., 2023). Among these isoenzymes, GSTP1 is the most extensively studied, with its transferase activity widely recognized as playing a pivotal role in drug resistance (Russell and Richardson, 2023; Russell et al., 2021). Notably, GSTP1 catalyzes the electrophilic conjugation of reduced glutathione with heterobiotic species and facilitates cell detoxification (Bartolini et al., 2019). Interestingly, GTSP1 has been implicated in conferring resistance to certain drugs, even though these drugs were not direct substrates of GSTP1. Consequently, further investigations suggested that, GSTP1 could be utilized to mediate tumor drug resistance through non-enzymatic pathways (Niitsu et al., 2022; Dong et al., 2019). For example, the antioxidant properties of GSTP1 inhibited oxidative stress damage caused by chemotherapy agents to protect cells from harm (Kamada et al., 2004; Singh et al., 2020; Russell and Richardson, 2023). Simultaneously, GSTP1 exists in both monomeric and dimeric forms, which are in dynamic equilibrium. As a monomer, GSTP1 binds to JNK to form a stable dimer complex (Li et al., 2016; Okamura et al., 2015), which maintains JNK in an inactive state and prevents transduction of the JNK downstream signaling pathway. Activation of the JNK pathway is considered to promote apoptosis (Liu and Lin, 2005). Thus, GSTP1 serves as an inhibitor of apoptosis triggered by chemotherapeutic agents. Given its dual role as a resistant enzyme and its signal-modulating function, GSTP1 can modulate drug efficacy regardless of whether the compound is a substrate for this enzyme.

Normal metabolic processes gives rise to ROS (Lushchak, 2014), which has a double-edged effect depending on its concentration (Huang and Li, 2020). Oxidative and antioxidant reactions exist in a dynamic equilibrium, with an optimal level of ROS being essential for normal cell growth and self-renewal. However, when this balance is disrupted due to various factors, excessive ROS levels may destroy the cellular proteins and nucleic acids through multiple mechanisms, resulting in cell damage (Harris and DeNicola, 2020). Currently, numerous chemotherapeutic agents such as cytarabine (Chiou et al., 2023), 5-azacitidine (Jin et al., 2020), imatinib, rituximab, arsenic trioxide, and doxorubicin (Perillo et al., 2020), were known to inhibit cell proliferation and induce apoptosis by triggering ROS levels. Studies have indicated that anthracyclines, like daunorubicin evoked ROS production in target cells (Stěrba et al., 2013). In our investigation, we demonstrated that DNR played a role in inducing cell death by promoting oxidative stress levels, whereas SPAG6 overexpression reduced this process. Furthermore, we also discovered that SPAG6 overexpression increased GSTP1 expression. As an antioxidant enzyme, GSTP1 improved the antioxidant capacity of cells and reduced DNR-induced oxidative stress. Simultaneously, the formation of the GSTP1-JNK complex was enhanced, which prevented activation of the JNK pathway and decreased the expression of p-JNK. Consequently, the transduction of the pro-apoptotic signaling pathway has been blocked, resulting in a reduction in apoptosis and ultimately diminishing the efficacy of DNR.

This study presented certain limitations. *In vivo*, the subcutaneous tumorigenesis experiment we conducted provides insights into the role of SPAG6 in tumor growth in mice to a certain extent; however, the mechanism through which SPAG6 regulates the progression of AML in this model requires further experimental investigation. Simultaneously, due to the challenges associated with culture and transfection of primary cells, we currently lack the data to support the effect of SPAG6 on DNR drug efficacy in primary cells. Additionally, we found that SPAG6 regulated GSTP1 expression; however, the specific dynamic process remains unclear and demands further experimental exploration.

We demonstrated a novel pathway through which SPAG6 affects drug efficacy: namely, the downregulation of SPAG6 leads to the activation of JNK pathway, elevates oxidative stress levels by reducing GSTP1 expression, and subsequently enhances DNR-induced apoptosis. Hence, SPAG6 knockdown may synergistically enhance the efficacy of chemotherapeutic drugs or reduce the dose of these drugs to mitigate their side effects, potentially offering a strategy for the treatment of AML.

## Data Availability

The datasets presented in this study can be found in online repositories. Further inquiries can be directed to the corresponding author.
